# Teeth during Pregnancy

**Published:** 1904-07

**Authors:** Thomas J. Giblin

**Affiliations:** Boston, Mass.


### TEETH DURING PREGNANCY.[1]

BY THOMAS J. GIBLIN, D.M.D., BOSTON, MASS.

You are to have an opportunity later in the evening to learn more about the very interesting subject of Orthodontia from one who is not always with you. That you may not be held too long in suspense I shall make my paper brief and touch upon only a few of the topics that my subject suggests.

Some eighteen years ago a woman, accompanied by her husband, visited my office. She was suffering intensely from periodontitis about an upper bicuspid. The husband told me his wife was pregnant about seven months, which was obvious. It was her intention, as soon as she was able, to have her upper teeth removed for the purpose of inserting an artificial denture, but just at that time she desired to have the troublesome tooth extracted. Her physician had told her to have it done if her dentist considered it would be right. As had been my practice for a few years previously, I administered gas and removed the tooth. Some time after the husband called at my office and in a disagreeable manner said, "Well, what are you going to do about my wife's illness?" For the first time I learned that his wife had had a miscarriage, was attended by several physicians and two nurses, and had passed through a very severe and critical experience. I was a young man, about to be married, and with much at stake, and the picture I conjured of law-suits, damages, and deterrent advertising was horrible. I insisted that I had used due care and had followed the usual practice in what I had done; that I was not responsible for his wife's illness, and consequently would not be responsible for the expense attending it.

I maintained that the remedies she had used and the long suffering she had endured previous to my attendance would account for the result. However, rather than at that time face the disagreeable alternative of the law-court, through my attorney I compromised with him and settled the case.

About two months ago I filled with gold a labial cavity in a lower cuspid for a young woman. The excavation was difficult, the tooth very sensitive, and the patient extremely nervous. She

[1] Read before the Harvard Odontological Society, May 26, 1904.

started at the appearance of the excavator or the bur. However, the filling was finally inserted, another appointment was made for further work, and the patient was dismissed. The subsequent appointment was not kept. After a few weeks she called and informed me that she had been ill; that she had had a miscarriage, and had been critically ill. Her physician ascribed as the cause the dental operation of a few days previous. As I had no knowledge of her condition, which was only about ten weeks' pregnancy, I was not held responsible.

These reminiscences have prompted me to offer this caution to young men for pregnant women,—defer all but absolutely necessary operation until the patient is in a more favorable condition. Use soothing, palliative measures, temporary fillings, etc. I have asked several students from different colleges how they were instructed, and found that it was taught that pregnancy is not a valid objection to extraction when extraction is advisable.

I do not believe that specific rules are possible, either in medicine or dentistry, to deal with the intricate and varying problems, and I respectfully dissent from the inference that it makes no difference whether or not a woman is pregnant in performing dental operations. The intimate relation between mother and child should be borne in mind. Whatever of physical or mental character affecting the mother may also affect the child. Causes of miscarriage may be violence, idiosyncrasy, disease of general or local character, reflex influence, mental, moral, or nervous, whether upon the uterine contractions or indirectly through the circulation upon the fœtus. That dental operations upon pregnant women may be the exciting cause of miscarriage I am fully convinced, and I have made it my practice, when I am aware of such condition, to do only such work as will prevent or relieve pain, and to carefully avoid nervous shock.

Passing from this topic, let us for a few minutes consider another.

It is a generally accepted fact that the teeth during pregnancy are very often attacked by rapidly developing caries. It has often been my experience to have been startled by destruction that has gone on during a comparatively short time upon the teeth of some woman whom I had seen previous to and subsequent to pregnancy. I have no doubt that you have each one had similar experience.

How can it be accounted for, and what preventive measures may be suggested?

Not so very long ago it was taught that during the growth of the fœtus the mother was called on to supply through the circulation of the blood the necessary lime salts; if the ingesta failed in supply, the bones of the mother, through the process of absorption, were called on to supply the deficiency. Thus it was supposed that even the teeth were rendered soft and readily susceptible to decay. This theory has been shown to be erroneous, because the teeth are not supplied with any system of absorbents, whereby the lime salts can be abstracted.

Even the bones, which are supplied with lymphatics, give no evidence that absorption goes on during this period.

As a matter of fact, the teeth once formed are least liable, of all the tissues of the body, to undergo changes dependent upon nutrition. While the theory was in vogue we frequently recommended an increased phosphate diet, in the form of certain kinds of food which contained an abundance of lime and magnesium salts, and even in the administration of the hypophosphites. The result was that we so overfed the mother that many cases of parturition were made difficult by the advanced calcification of the fœtus.

The conclusion of empiricism—and most of the conclusions of medicine are empiric—is that the mother normally ingests sufficient phosphates for the necessary work of gestation.

It is much more rational to explain the ravages upon the teeth during pregnancy by directing our search and examination of the environment of the teeth during gestation. We are all convinced of the theory of caries as advanced by Miller,—lactic acid and bacteria, the lactic acid acting upon the tooth and, later, furnishing soil for the culture of bacteria.

I made a request of the house physician of one of our lying-in hospitals here in Boston to make certain tests for me. He reported to me yesterday that he examined the mouths of twenty pregnant women, of from five to nine months, and in every case found the saliva strongly acid, in both the morning and evening tests.

Here we have ready complete and sufficient explanation of the cause of rapid caries during pregnancy. If we add to this cause the action of the acid of the stomach following the "morning sickness" and the frequent neglect of the usual care of teeth by women in this condition, we no longer wonder at the destruction we are so often called upon to repair.

If you agree with me in the conclusion at which I have arrived,—that during pregnancy the environment of the teeth is dangerous to the integrity of that organ,—how shall we best prevent the harm?

By instructing those patients where possible that exceptional care must be exercised during that period, and in particular calling the attention of a physician, friend, or patient to the facts I have laid before you, and urge him to prescribe to every pregnant woman an alkaline mouth-wash as a *sine qua non.*

